# Recovery after spinal cord relapse in multiple sclerosis is predicted by radial diffusivity

**DOI:** 10.1177/1352458510376180

**Published:** 2010-10

**Authors:** Patrick Freund, Claudia Wheeler-Kingshott, Jonathan Jackson, David Miller, Alan Thompson, Olga Ciccarelli

**Affiliations:** 1 Department of Brain Repair and Rehabilitation, NMR Unit, UCL Institute of Neurology, London, UK.; 2 Wellcome Trust Centre for Neuroimaging, UCL Institute of Neurology, London, UK.; 3 Department of Neuroinflammation, NMR Unit, UCL Institute of Neurology, London, UK.

**Keywords:** Disability, multiple sclerosis, recovery, repair, spinal cord

## Abstract

**Background:** The aim of this study was to determine whether the diffusion tensor-derived radial diffusivity and axial diffusivity, measured in the cortico-spinal tract in the cervical cord, predict clinical recovery after a cord relapse in patients with multiple sclerosis, and change over time.

**Methods:** Fourteen patients were clinically assessed at the onset of a cervical cord relapse and after 1, 3 and 6 months. Patients and 13 age-matched healthy controls underwent spinal cord diffusion tensor imaging at each time point. The directional diffusivities from diffusion tensor imaging, termed radial diffusivity and axial diffusivity, were calculated in regions of interest placed in the lateral columns, where the cortico-spinal tract is located, and in the anterior and posterior columns. Regression analyses identified predictors of clinical outcome, adjusting for age, gender, cord cross-sectional area and baseline clinical score, and estimated the differences in the rate of change in diffusion tensor imaging measures between groups over time, adjusting for changes in cord cross-sectional area.

**Results:** Lower radial diffusivity of the cortico-spinal tract at baseline was associated with better clinical outcome. As patients improved clinically during the follow-up, they showed greater decrease in radial diffusivity of the cortico-spinal tract than controls.

**Conclusions:** The predictive role of radial diffusivity and its dynamic changes over time suggest that this index reflects spinal cord pathological processes, including resolution of inflammation and remyelination, that contribute to clinical recovery in multiple sclerosis. This suggests that radial diffusivity may be useful in trials that promote recovery after spinal cord injury and could be applied to other neurological diseases affecting the spinal cord.

## Introduction

Relapses are a distinctive feature of multiple sclerosis (MS) and are associated with new inflammatory lesions on magnetic resonance imaging (MRI).^1^ New lesions in the spinal cord can cause disabling symptoms, because important neurological functions are conveyed within a narrow canal space. Although the majority of patients make a clinical recovery, in some cases recovery is only partial, leading to accumulation of disability. Understanding the neurobiological mechanisms behind this incomplete (and occasionally absent) clinical recovery is essential in order to develop effective treatments that reduce the impact of acute injury to the spinal cord.

Here we investigated changes in the microstructure of the cervical cord affected by an acute lesion, in patients with MS, by measuring the spinal cord directional diffusivities, which are derived from the diffusion tensor (DT),^2^ and which have the potential to reflect pathological processes^3^^–^^6^ more accurately than standard DT imaging (DTI) indices. The directional diffusivities are: (1) the ‘axial diffusivity’ (AD), which is the principal eigenvalue of the DT, and (2) the ‘radial diffusivity’ (RD), which is the average of the second and third eigenvalues of the DT. AD is considered to represent the water diffusion parallel to the main structure of the voxel, e.g. white matter fibres,^7^ whilst the RD represents the water diffusion perpendicular to this direction, hence to the white matter fibres. In the spinal cord of mice with experimental allergic encephalomyelitis (EAE), decreased AD and increased RD are markers of axonal damage and demyelination, respectively.^8^^,^^9^

In a previous study in which we examined the directional diffusivities in the spinal cord of patients with MS at the onset of a relapse due to a lesion at C1–C3,^10^ we found that the RD of the lateral cortico-spinal tract (CST) essentially drove the observed changes in fractional anisotropy (FA), which is a standard DTI-derived index obtained by combining the three eigenvalues of the DT; RD also showed a stronger correlation with acute disability than did FA. However, a recent DTI study of the optic nerve found that AD decreased acutely after optic neuritis, and was reported to be a useful predictor of vision.^11^

Therefore, in order to determine whether AD and RD at the onset of an acute event predict subsequent clinical recovery, we carried out a prospective longitudinal study in the same cohort of MS patients, and employed spinal cord DTI and a region-of-interest (ROI) analysis. In particular, we tested two hypotheses: (1) RD and AD of the CST in the lateral columns predict clinical recovery; and (2) RD and AD of the CST change over time in patients when compared with controls. To complete the analysis, we also investigated the standard diffusion indices, such as FA, and measured the directional diffusivities and the standard DTI indices in the anterior and posterior spinal cord columns.

## Methods

### Subjects

We recruited patients who attended the Outpatient Clinic at the National Hospital for Neurology and Neurosurgery (NHNN) and fulfilled the following inclusion criteria: (1) a diagnosis of MS;^12^ (2) acute development of motor signs, which were judged to be due to a lesion at C1–C3; (3) onset within 4 weeks of visit; and (4) presence of at least one lesion in the spine between C1 and C3 documented by conventional imaging. Due to radiological characteristics and clinical signs, despite the absence of contrast-enhanced imaging, these cervical lesions were considered to be very likely acute, as previously explained.^10^

Patients were invited to attend follow-up visits at 1, 3 and 6 months. At each visit, patients underwent MRI scans, and were clinically assessed on the Expanded Disability Status Scale (EDSS),^13^ 9-Hole Peg Test (9HPT),^14^ Timed 25-foot Walk Test (TWT),^15^ and Multiple Sclerosis Walking Scale-12 (MSWS-12).^16^ The average of two trials for each hand of the 9HPT and the inverse of the mean of two trials for the TWT (iTWT) were calculated.^17^

Age- and gender-matched healthy subjects were recruited at baseline and invited to attend MRI scans at the same time points.

All subjects gave informed, written consent before the study, which was approved by the Joint Ethics Committee of the Institute of Neurology and the NHNN.

### MRI protocol and processing

MRI of the cervical cord was performed on a 1.5T GE scanner (maximum gradient amplitude =33 × 10^−3^ Tm^−1^). At each time point, all subjects were repositioned in the scanner in a consistent way by the radiographer and underwent the following spinal cord sequences:

- T2 and PD-weighted imaging (TR 3300 ms/TE 110 ms and TR 3000ms/TE 8.9 ms, echo train length 33 and 11, FOV 240 × 240mm^2^, matrix 256 × 224, interpolated to 512 × 512, 12 contiguous sagittal- and coronal-oblique slices, 3 mm slice thickness), which was used to confirm the presence of the lesion at C1–C3 and screen for cord swelling (as defined in Ciccarelli et al.^10^) at each time point.
- Cardiac gated CO-ZOOM-EPI (contiguous zonally orthogonal multislice echo-planar imaging)^18^ (TE 96 ms, TR ≈ 15 RR, six non-diffusion-weighted images, diffusion gradients applied along 60 optimized diffusion weighting directions,^19^ maximum b-factor of 1000 s/mm^2^, FOV 70 × 47 mm^2^, matrix 48 × 32, reconstructed to 1 × 1 mm^2^, 30 contiguous axial slices, 5 mm slice thickness).
- Volume-acquired, inversion-prepared, fast-spoiled-gradient recalled (FSPGR) imaging (TR 13.2 ms, TE 4.2 ms, TI 450 ms, flip angle 20°, FOV 250 × 250 mm^2^, matrix 256 × 256, 60 contiguous sagittal slices, 1 mm slice thickness), which was used to calculate the cross-sectional cord area at C2–C3 using a semi-automated technique^20^ at each time point.

### Diffusion tensor calculation and region-of-interest analysis

The DT data were screened manually for slices affected by artefacts, which were marked for exclusion from the tensor fitting. An anisotropic diffusion filter was applied to the DTI data to reduce image noise.^21^ RD, AD, FA (which reflects the directionality of the underlying tissue structures), and mean diffusivity (MD) (which reflects the amount of water diffusion regardless of its preferential directionality) maps were obtained from the DT estimated with the RESTORE algorithm (robust estimation of diffusion tensor with outlier rejection^22^), using Camino software.^22^^,^^23^ A condition for the use of RESTORE is that the percentage of excluded artifactual data points is low (≤6.67%).^22^ Therefore, datasets with ≥6% (or ≥4) diffusion-weighted volumes affected by artefacts were excluded from the analysis. The directionality of the principal eigenvalue compared with the spinal cord anatomical structures was visually checked in each subject at each time point.

**Figure 1.**
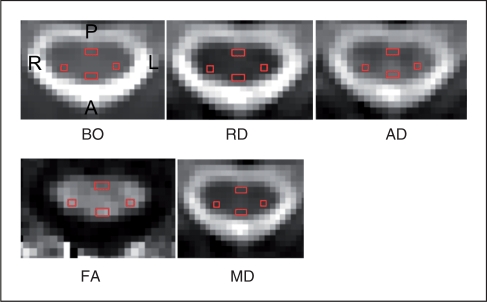
Axial images (B0, RD, AD, FA, MD) of the cervical spinal cord showing the location of regions of interest (ROIs) placed in the anterior, lateral and posterior columns of the spinal cord. Note that ROI were first drawn on the B0 images and then overlaid onto the diffusion maps. (A = anterior, P = posterior, L = left, R = right). AD, axial diffusivity; FA, fractional anisotropy; MD, mean diffusivity; RD, radial diffusivity.

In each subject, at each time point, four ROIs were placed on each non-diffusion-weighted b0 image between C1 to C3. They were located in the posterior part of the lateral columns, where the lateral CST is known to run through (ROI size = 4 voxels (24 mm^3^)), and in the anterior and posterior columns (ROI size = 6 voxels (36 mm^3^)) (Figure 1). As it is impossible to distinguish lesions from normal tissue on the b0 maps, these ROIs probably encompassed not only the focal lesions, but also the non-lesional white matter. The ROIs were copied directly onto the derived RD, AD, FA, and MD maps (Figure 1). The mean value of each diffusion parameter within each ROI was calculated. To obtain the intra-observer coefficient-of-variation (CV = (SD/mean) × 100) for the DTI indices in each ROI, a second experiment was run by the same observer (PF), who was blind to the results of the first experiment, 3 months later, on three patients and three controls randomly chosen. The mean CV of the diffusion indices of all ROIs was 2.7% (Supplementary Table 1).

**Table 1.** Clinical scores at baseline and 6 months follow-up

	Baseline	6 months	*p*-value*
EDSS	Median 4 (range 2.5, 6.5)	Median 3.5 (range 1, 6.5)	0.005
TWT (s)	Mean 9 (SD 3.2)	Mean 7 (SD 1.2)	<0.0001
MSWS-12	Mean 47 (SD 7)	Mean 33 (SD 13)	<0.0002
9HPT (s)	Mean 36.8 (SD 37.6)	Mean 23.02 (SD 3.31)	0.184

* paired *t*-test.

EDSS, Expanded Disability Status Scale; MSWS-12, Multiple Sclerosis Walking Scale-12; TWT, Timed 25-foot Walk Test; 9HPT, 9-Hole Peg Test.

### Statistical analysis

#### Changes in clinical scores over the follow-up period

To assess whether patients’ clinical scores changed significantly from baseline to 6 months, the paired sample *t*-test was used (after checking the normal distribution of the scores).

#### Differences in diffusion indices between patients and controls at each time point

At each time point, to investigate the differences in each diffusion index of each ROI between patients and controls, the two-sample *t*-test was used.

#### Baseline predictors of outcome

To identify independent predictors of clinical changes in patients, a multiple linear regression model was used. ITWT, 9HPT, MSWS-12 at 6 months were used, in turn, as dependent variable. The baseline diffusion indices were used as covariates, together with the corresponding baseline clinical score, age, gender, and baseline cord cross-sectional area.

For the analysis of the EDSS, an ordinal logistic multiple regression model was used, entering an indicator of the clinical outcome, defined on the basis of the EDSS changes, as response variable (i.e. the ‘presence of clinical improvement’ was defined as a reduction in the EDSS between 6 months and baseline ≥−0.5, and the ‘absence of recovery’ was defined as the lack of any EDSS change). The baseline diffusion indices were used as covariates, together with baseline EDSS, age, gender, and baseline cord cross-sectional area. Using mixed-effect linear regression models, we also investigated whether the diffusion measures at baseline differed between patients who improved and patients who did not recover, using the diffusion index as response variable, and entering an indicator for these two groups of patients, and all the variables listed above, as covariates. For all these regression models, the mean values of the diffusion indices of the left and right CST-ROI were used, since there were no significant differences in any baseline DTI parameter between the left and right side in patients and controls (using the Wilcoxon-Signed Rank test).

#### Longitudinal analysis: Estimation of the rate of change in the diffusion indices over time

Mixed-effect linear regression models were used to estimate the rate of change in the DTI indices over time, adjusting for age and changes in cord cross-sectional area, which has been shown to decrease over time in this patient cohort.^24^ In particular, after confirming that time delay from onset of symptoms to baseline MRI did not influence the diffusion measures at study entry, each DTI measure of each ROI was used, in turn, as response variable, with time as predictor. A subject-type indicator and type × time interaction term were used to estimate the difference in the rate of changes between patients and controls, and to assess the rate of changes in controls. These models allow for missing time points, and maximize efficient use of the available data.

Stata 9.2 (www.stata.com) was used. Results associated with *p* < 0.05 are reported.

## Results

### Subjects

Fourteen MS patients were recruited at baseline (mean age 35.3 years (SD 8.16), nine women, 13 patients with relapsing-remitting and one with secondary-progressive MS). One patient did not undergo any follow-up scan, but was clinically assessed at 6 months; one patient did not attend his 1-month follow-up visit, and the DTI was not acquired in one patient at 1-month follow-up, due to technical problems with the scanner. Details relating to patient symptoms and conventional MRI characteristics, including T2 lesions and cord swelling at baseline and at 6 months, have been reported previously;^10^ baseline cervical cord swelling resolved within a month in all cases except one.

All 13 age- and gender-matched controls (mean age 40.9 years (SD12.6), eight women; two-sample *t*-test (controls vs. patients) *p* = 0.21) studied at baseline attended their follow-up scans.

### Changes in clinical scores over the follow-up period

Patients improved over time, as reflected in changes in their EDSS, iTWT and MSWS-12 scores (Table 1). Nine patients improved on the EDSS and five patients did not.

### Differences in diffusion indices between patients and controls at each time point

A total of 24, 20, 19 and 21 datasets at baseline, 1, 3 and 6 months, respectively, were included in the final analysis. Patients showed higher RD in the right and left CST-ROI at baseline, in the right CST-ROI at 6 months, and in the anterior and posterior columns at all time points; AD in the posterior columns differed between patients and controls at three months (Table 2).

**Table 2.** Values of the diffusion indices obtained in the cervical spinal cord in regions of interest in the left and right lateral cortico-spinal tract, anterior and posterior columns, at each time point, in patients with multiple sclerosis and controls

	Baseline			1 Month			3 Month			6 Month		
	13 Pt.	11 Co.		9 Pt.	11 Co.		10 Pt.	9 Co.		11 Pt.	10 Co.	
	Mean (SD)	Mean (SD)	P-value	Mean (SD)	Mean (SD)	P-value	Mean (SD)	Mean (SD)	P-value	Mean (SD)	Mean (SD)	P-value
**RD** mm^2^/s × 10^−3^
L-CST	**0.54 (0.12)**	**0.41 (0.01)**	**0.01**	0.48 (0.16)	0.41 (0.06)	n.s.	0.42 (0.1)	0.44 (0.11)	n.s.	0.48 (0.09)	0.44 (0.07)	n.s.
R-CST	**0.55 (0.15)**	**0.44 (0.13)**	**0.05**	0.54 (0.18)	0.45 (0.07)	n.s.	0.51 (0.15)	0.43 (0.15)	n.s.	**0.52 (0.12)**	**0.42 (0.09)**	**<0.05**
PC	**0.49 (0.11)**	**0.4 (0.09)**	**0.01**	**0.52 (0.11)**	**0.41 (0.05)**	**<0.01**	**0.6 (0.11)**	**0.36 (0.09)**	**<0.001**	**0.53 (0.15)**	**0.4 (0.08)**	**<0.01**
AC	**0.67 (0.15)**	**0.41 (0.14)**	**<0.001**	**0.62 (0.19)**	**0.40 (0.07)**	**<0.05**	**0.62 (0.2)**	**0.38 (0.11)**	**<0.01**	**0.56 (0.14)**	**0.35 (0.07)**	**<0.05**
**AD** mm^2^/s × 10^−3^												
L-CST	1.11 (0.12)	1.14 (0.23)	n.s.	1.06 (0.14)	1.15 (0.14)	n.s.	1.04 (0.2)	1.12 (0.08)	n.s.	1.05 (0.2)	1.0 (0.19)	n.s.
R-CST	1.12 (0.14)	1.14 (0.23)	n.s.	1.12 (0.17)	1.17 (0.18)	n.s.	1.1 (0.2)	1.1 (0.17)	n.s.	1.06 (0.23)	1.03 (0.18)	n.s.
PC	0.98 (0.15)	0.97 (0.21)	n.s.	1.03 (0.15)	1.01 (0.12)	n.s.	**1.09 (0.13)**	**0.94 (0.13)**	**<0.05**	0.96 (0.21)	0.92 (0.2)	n.s.
AC	1.13 (0.1)	1.17 (0.25)	n.s.	1.24 (0.19)	1.21 (0.14)	n.s.	1.2 (0.23)	1.18 (0.17)	n.s.	1.09 (0.28)	1.05 (0.22)	n.s.
**FA**												
L-CST	**0.45 (0.1)**	**0.58 (0.07)**	**0.001**	0.51 (0.06)	0.58 (0.06)	0.08	0.54 (0.09)	0.55 (0.07)	n.s.	0.48 (0.08)	0.52 (0.04)	n.s.
R-CST	**0.45 (0.1)**	**0.57 (0.07)**	**<0.01**	0.48 (0.09)	0.55 (0.06)	0.08	0.49 (0.11)	0.55 (0.08)	n.s.	**0.44 (0.06)**	**0.53 (0.04)**	**<0.001**
PC	**0.4 (0.08)**	**0.55 (0.05)**	**<0.001**	**0.44 (0.08)**	**0.52 (0.09)**	**<0.01**	**0.39 (0.07)**	**0.53 (0.08)**	**<0.01**	**0.41 (0.08)**	**0.50 (0.06)**	**<0.01**
AC	**0.35 (0.1)**	**0.61 (0.09)**	**<0.001**	**0.44 (0.13)**	**0.63 (0.06)**	**<0.01**	**0.44 (0.11)**	**0.64 (0.09)**	**<0.001**	**0.44 (0.1)**	**0.61 (0.06)**	**<0.001**
**MD** mm^2^/s × 10^−3^												
L-CST	0.73 (0.11)	0.65 (0.13)	n.s.	0.64 (0.06)	0.66 (0.07)	n.s.	0.63 (0.13)	0.66 (0.12)	n.s.	0.67 (0.12)	0.62 (0.1)	n.s.
R-CST	0.74 (0.13)	0.67 (0.14)	n.s.	0.75 (0.11)	0.68 (0.09)	n.s.	0.71 (0.15)	0.67 (0.14)	n.s.	0.7 (0.15)	0.61 (0.11)	**<0.05**
PC	0.65 (0.12)	0.57 (0.13)	n.s.	**0.67 (0.1)**	**0.6 (0.06)**	**<0.05**	**0.77 (0.12)**	**0.57 (0.09)**	**<0.05**	**0.69 (0.16)**	**0.57 (0.12)**	**<0.01**
AC	**0.86 (0.14)**	**0.67 (0.17)**	**<0.01**	**0.79 (0.12)**	**0.66 (0.06)**	**<0.05**	**0.81 (0.2)**	**0.64 (0.11)**	**<0.001**	**0.71 (0.17)**	**0.6 (0.11)**	**<0.05**

L, left; R, right; CST, cortico-spinal tract; PC, posterior columns; AC, anterior columns; Pt., patients; Co., controls; RD, radial diffusivity; AD, axial diffusivity; FA, fractional anisotropy; MD, mean diffusivity.

Turning to the standard diffusion indices, patients had lower FA than controls in the right and left CST-ROI at baseline, in the right CST-ROI at 6 months, and in the anterior and posterior columns at all time points. MD in the posterior columns was significantly higher in patients than in controls at 1 and 3 months, whereas MD in the anterior columns was higher in patients than controls at baseline, 1 and 3 months (Table 2).

### Baseline predictors of recovery

**Figure 2.**
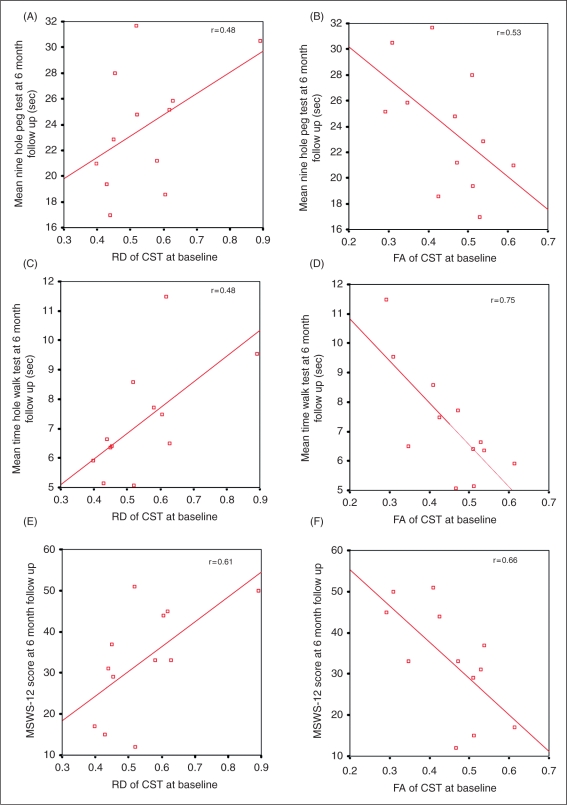
Graphs showing the correlations between the diffusion indices at baseline and clinical score at 6 months: (A) RD of the CST-ROI vs. 9HPT; (B) FA of the CST-ROI vs. 9HPT; (C) RD of the CST-ROI vs. TWT; (D) FA of the CST-ROI vs. TWT; (E) RD of the CST-ROI vs. 12-MSWS; (F) FA of the CST-ROI vs. MSWS-12. Fitted lines and *R*^2^ are shown. CST, cortico-spinal tract; FA, fractional anisotropy; TWT, Timed 25-foot Walk Test; 9HPT, 9-Hole Peg Test; MSWS-12, Multiple Sclerosis Walking Scale-12.

Lower RD of the mean of the left and right CST-ROI at baseline predicted better outcome; in particular, baseline RD was associated with iTWT (coeff. −1.48 × 10^−4^, *p* = 0.026, 95% confidence interval (CI) −2.73 × 10^−4^, 2.2 × 10^−5^) and 9HPT (coeff. 0.02, *p* = 0.041, 95% CI 0.001, 0.04) at 6 months, and showed a borderline association with MSWS-12 (coeff. 0.06, *p* = 0.07; 95% CI −0.006, 0.12) at 6 months (Figure 2).

When we examined the standard diffusion indices, we found that higher FA of the mean lateral CST-ROI also predicted better outcome; in particular, baseline FA was associated with iTWT (coeff. 2.2 × 10^−5^, *p* = 0.014, 95% CI 6 × 10^−6^, 3.8 × 10^−5^), 9HPT (coeff. −2.84 × 10^−3^, *p* = 0.032, 95% CI −5.38 × 10^−3^, −3.03 × 10^−4^) and MSWS-12 (coeff. −8.69 × 10^−3^, *p* = 0.045, 95% CI −0.02, −2.18 × 10^−4^) at 6 months (Figure 2).

Patients who improved on the EDSS showed a significantly lower RD of the mean lateral CST-ROI at baseline, and a significantly higher FA of the same tract at baseline, when compared with patients who did not recover (RD of the CST-ROI: patients who improved/patients who did not recover, difference −0.13, *p* = 0.018, 95% CI −0.24, −0.02; FA of the CST-ROI: 0.09, *p* = 0.016, 95% CI 0.02, 0.16).

### Longitudinal analysis: estimation of the rate of change in the diffusion indices over time

**Figure 3.**
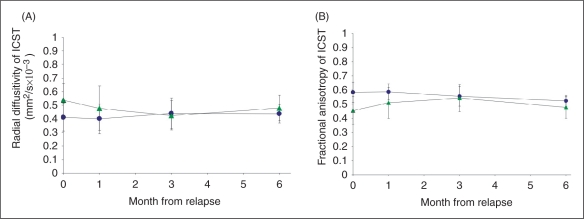
Mean values (and SDs) of the radial diffusivity (A) and fractional anisotropy (B) of the left cortico-spinal tract (lCST) in MS patients (in green) and controls (in blue) at baseline, 1 month, 3 months and 6 months.

Patients showed a greater decline in the RD of the left CST-ROI over the follow-up period when compared with controls (by 0.016 mm^2^/s × 10^−3^ per month, *p* = 0.049, 95% CI 6 × 10^−5^, 0.031), adjusting for changes in cord cross-sectional area over time (Figure 3). The rate of change in AD of this tract did not significantly differ between patients and controls (by 0.011 mm^2^/s × 10^−3^ per month, *p* = 0.382, 95% CI −0.036, 0.013).

Turning to the conventional diffusion indices, patients showed a greater increase in the FA of the left lateral CST-ROI compared with controls over time (by 0.01 mm^2^/s × 10^−3^ per month, *p* = 0.027, 95% CI 0.24, 0.01). The rate of change in MD of this tract did not differ between groups (by 0.007 mm^2^/s × 10^−3^ per month, *p* = 0.393, 95% CI −0.01, 0.025).

In controls, no diffusion index in any region changed over time.

## Discussion

Animal studies have demonstrated (i) the potential of the directional diffusivities to provide information on the pathological processes in addition to that derived from the conventional diffusion indices,^3^^–^^6^^,^^8^^,^^9^^,^^25^^–^^27^ and (ii) their sensitivity to pathological changes in the spinal cord at the site of the focal lesions and in locations distal to them.^28^ Our study in patients with MS after an upper cervical cord relapse suggests that RD is sensitive to tissue microstructural changes that influence clinical recovery and are dynamic over time. In particular, we found that the RD of the CST-ROI, which encompassed the lesional and non-lesional tissues, predicted a better clinical outcome after 6 months, and significantly decreased over time. These results were significant after correcting for cord cross-sectional area which, in the acute phase, is an indirect measure of the amount of oedema related to acute inflammation (i.e. cord swelling), while in the chronic phase is a marker of cord axonal loss.^29^

From a pathophysiological perspective, several mechanisms may contribute to tissue repair and consequent clinical improvement. In a previous study carried out in the same patient population, we proposed that increased mitochondrial metabolism contributes to clinical recovery.^24^ In addition to this repair mechanism, resolution of inflammation and remyelination also occur. Remyelination, in particular, leads to the restoration of myelin sheaths on demyelinated axons which, in turn, re-establishes saltatory conduction and resolves symptoms of acute relapse.^30^ Although linking RD to myelin integrity in the spinal cord of these patients is tempting, especially considering the extensive body of evidence from animal studies,^5^ it is likely to be an oversimplification of the underlying tissue changes. Indeed, robust RD changes can be seen in the absence of any myelin loss,^6^ and in vivo brain studies in patients with MS have suggested that the radial component of the DT does not selectively measure demyelination.^31^ Further imaging studies, which will combine different indices sensitive to myelin and axonal integrity, together with histological findings, should confirm the relative specificity of the AD and RD to acute and chronic demyelination and axonal loss in the human spinal cord.

From a technical point of view, we chose an ROI approach in order to be confident that the ROIs are positioned in the columns that contain ascending and descending tracts. In these regions, the directional diffusivities reflect the underlying structural characteristics.^32^ An alternative methodological approach would be to employ diffusion-based tractography, as reported in our previous publications in MS^10^ and in other neurological disorders.^33^ However, in humans, the exact correspondence between the reconstructed tracts and the real pathways cannot be confirmed as convincingly as in animals,^28^ and ROIs need to be used in order to seed the tracts. In addition, since the diffusion parameters in the left CST tends to have greater FA values than the right CST in healthy subjects,^34^ and MS pathology may be asymmetric in the spinal cord, we kept the division between the left and right CST in the analysis of the comparisons between patients and control.

Moreover, we could not investigate the association between lesion location and the diffusion indices because it was difficult to locate the lesions on the basis of the sole sagittal images, and the differentiation of lesional tissue from the normal-appearing white matter was not possible on the diffusion maps.

The results relating to AD were negative, suggesting that this index may be less sensitive than RD in detecting spinal cord pathological changes occurring in the spinal cord after acute lesions. Conversely, a recent DTI study proposed AD as a marker of acute axonal injury,^27^ and another study in patients with optic neuritis reported that this index was associated with tests of vision after 1 and 3 months.^11^ However, the same authors found that RD, in contrast to AD, showed the highest correlations with clinical tests when patients with well-established disease were studied.^11^ Their hypothesis was that in the presence of extensive demyelination, AD may lose its sensitivity to reflect axonal integrity. However, in an animal model of EAE, AD was found to correlate with acute axonal injury.^27^ We cannot exclude that a constant AD is still compatible with axonal loss, if the larger diameter fibres are preserved and the fibres of small diameter of the CST are preferentially lost.^35^ However, the interpretation of AD and RD in relation to pathological changes and observations in animal studies is likely to be an oversimplification in respect to what is happening in MS, where complex tissue changes are occurring; for example, both increased^36^ and reduced AD^11^^,^^31^ has been described in MS and optic neuritis patients when compared with controls.

The behaviour of the diffusion indices varied according to the regions investigated. For example, the FA and RD of the lateral CST-ROI differed between patients and controls at baseline (also when adjusting for delay from symptom onset, results not shown), but then normalized over time, whilst the same diffusion measures in the anterior and posterior columns were abnormal in patients at all time points. This could be explained by the specific location of the lesion in the spinal cord. Unfortunately, we could not investigate the association between lesion location (anterior, posterior, lateral, for example) and the diffusion indices, because the differentiation of lesional tissue from the normal-appearing white matter was not possible on the diffusion maps. However, all the lesions responsible for clinical signs were within the ROIs studied. Studies using high-resolution spinal cord imaging will assess the contribution of the lesion and that of the surrounding and connected white matter in determining clinical changes.

In conclusion, the RD of the CST-ROI in the region of the cord affected by an acute lesion predicts clinical outcome after a spinal cord relapse and reflects the dynamic pathological changes that influence recovery. Future studies will investigate whether the role of AD as predictor of outcome becomes more relevant in conditions which are characterized by predominant axonal degeneration. Thus, RD is an informative measure which may become useful in clinical trials that promote recovery after spinal cord acute lesions, once accurate standardization of protocol acquisition, including a specific b-value and pre-determined number of diffusion gradients directions and tensor fitting, is performed.^37^ Investigations of spinal cord RD deserves to be extended to other neurological diseases affecting the spinal cord.
